# Barriers and Facilitators of Artificial Intelligence in Family Medicine: An Empirical Study With Physicians in Saudi Arabia

**DOI:** 10.7759/cureus.49419

**Published:** 2023-11-26

**Authors:** Turki Alanzi, Raghad Alotaibi, Rahaf Alajmi, Zainab Bukhamsin, Khadija Fadaq, Nouf AlGhamdi, Norah Bu Khamsin, Lujain Alzahrani, Ruya Abdullah, Razan Alsayer, Afrah M Al Muarfaj, Nouf Alanzi

**Affiliations:** 1 Department of Health Information Management and Technology, College of Public Health, Imam Abdulrahman Bin Faisal University, Dammam, SAU; 2 Department of Family Medicine, King Fahad Medical City, Riyadh, SAU; 3 College of Medicine, Imam Abdulrahman Bin Faisal University, Dammam, SAU; 4 College of Clinical Pharmacy, Imam Abdulrahman Bin Faisal University, Dammam, SAU; 5 Faculty of Medicine, Vision Colleges, Riyadh, SAU; 6 College of Medicine, King Abdulaziz University, Jeddah, SAU; 7 Faculty of Medicine, Ibn Sina National College, Jeddah, SAU; 8 College of Medicine, Northern Border University, Arar, SAU; 9 Department of Health Affairs, General Directorate of Health Affairs in Assir Region, Ministry of Health, Abha, SAU; 10 Department of Clinical Laboratory Sciences, College of Applied Medical Sciences, Jouf University, Sakakah, SAU

**Keywords:** benefits, challenges, technology acceptance, cancer, virtual assistants, family medicine, artificial intelligence

## Abstract

Background: Artificial intelligence (AI) is a novel technology that has been widely acknowledged for its potential to improve the processes' efficiency across industries. However, its barriers and facilitators in healthcare are not completely understood due to its novel nature.

Study purpose: The purpose of this study is to explore the intricate landscape of AI use in family medicine, aiming to uncover the factors that either hinder or enable its successful adoption.

Methods: A cross-sectional survey design is adopted in this study. The questionnaire included 10 factors (performance expectancy, effort expectancy, social influence, facilitating conditions, behavioral intention, trust, perceived privacy risk, personal innovativeness, ethical concerns, and facilitators) affecting the acceptance of AI. A total of 157 family physicians participated in the online survey.

Results: Effort expectancy (μ = 3.85) and facilitating conditions (μ = 3.77) were identified to be strong influence factors. Access to data (μ = 4.33), increased computing power (μ = 3.92), and telemedicine (μ = 3.78) were identified as major facilitators; regulatory support (μ = 2.29) and interoperability standards (μ = 2.71) were identified as barriers along with privacy and ethical concerns. Younger individuals tend to have more positive attitudes and expectations toward AI-enabled assistants compared to older participants (p < .05). Perceived privacy risk is negatively correlated with all factors.

Conclusion: Although there are various barriers and concerns regarding the use of AI in healthcare, the preference for AI use in healthcare, especially family medicine, is increasing.

## Introduction

Artificial intelligence (AI) has emerged as a transformative force in modern healthcare, promising to revolutionize clinical decision-making, patient care, and practice efficiency [1]. In the realm of family medicine, where comprehensive and patient-centered care is paramount, the integration of AI holds significant potential. However, realizing the full benefits of AI adoption in this context necessitates a comprehensive understanding of the barriers and facilitators that shape its implementation [2-4]. AI in healthcare is believed to offer various benefits. AI-powered algorithms can analyze medical data, including medical images, genetic information, and patient records, to detect diseases, predict patient outcomes, and provide early diagnosis. This can lead to faster and more accurate diagnoses, improving patient outcomes. AI can enhance medical imaging, such as X-rays, MRI, and CT scans, by providing more accurate interpretations and identifying abnormalities that may be missed by human radiologists [5]. AI can tailor treatment plans to individual patients by analyzing their medical history, genetic makeup, and specific health conditions. This leads to more effective and efficient treatment options, minimizing trial-and-error approaches [6]. AI enables remote patient monitoring through wearable devices and telemedicine platforms. Patients can receive real-time health insights and access healthcare services from the comfort of their homes, improving access and reducing healthcare costs [7].

AI can optimize hospital and clinic operations by automating administrative tasks, managing patient schedules, and predicting patient admissions and discharges. This leads to improved resource allocation and cost savings [8]. AI systems are not prone to fatigue or distraction, reducing the likelihood of human errors in healthcare, which can have life-threatening consequences [6]. AI-driven chatbots and virtual assistants can interact with patients, answer their queries, and provide health information, improving patient engagement and education [9]. AI solutions can be deployed globally and are accessible even in remote areas, making healthcare services available to a broader population [9].

Empirical research on the acceptance of AI in healthcare has revealed various barriers and facilitators, whose impact is varied across the studies. A recent systematic review of 60 studies and an empirical study with 758 physicians and medical students from 39 countries has identified some interesting results [10]. Only 10-30% of the participants across the studies had used AI, and 74.28% (35 studies) suggested lack of AI knowledge among the physicians. In addition, the empirical study revealed 38% awareness rate, 20% utility rate, and 53% unawareness rate. In addition, risks such as inaccurate results were identified to be one of the major barriers to accepting AI. The study observed that, although physicians were aware of increasing application of AI in healthcare, they lacked essential practical and theoretical knowledge in using AI but held positive attitudes towards AI. Another study [11] focusing on the acceptance of AI among oncologists as an assistant technology in decision-making observed that factors including performance expectancy (PE: the level of individual believes that the use of the system can help him gain benefits in his activities), social influence (SI: an individual feels the importance that the others believe he or she should use the new system), and facilitating conditions (FC: the degree to which an individual believes that an organization’s and technical infrastructure exists to support the use of the system) [12] significantly affected the oncologist's behavioral intentions (BI) to use AI. However, effort expectancy (EE: the level of ease of use associated with the use of a system), and perceived risks (uncertainty that consumers experience when they cannot foresee the consequences of using a system) did not significantly affect BI [12]. In a similar study investigating the prospects of using AI-based decision-making among physicians, EE and SI were positively correlated with trust (a willingness to depend on the specific technology in a given situation in which negative consequences are possible), and SI was positively correlated with BI, and no association was observed between PE and trust [13]. Similarly, in another study focusing on dental healthcare workers, PE and EE were positively correlated with BI; SI and trust mediated the relationship between PE and BI; and SI and trust mediated the relationship between EE and BI. In another study, physicians' perceptions and EE were negatively correlated with risks, indicating that perceived risks can indirectly affect the intentions to use AI [14]. Furthermore, personal innovativeness (the individual's propensity and willingness to explore and examine new technologies and innovations) was observed to have significant positive impact on the BI to use AI-based virtual assistants in a recent study [15]. These findings indicate that the impact of different factors on the BI to use AI in healthcare varied across the participants, indicating the need to further explore the area in different settings to gain a better understanding of the acceptance of AI in healthcare.

Considering this aspect, this study explores the intricate landscape of AI use in family medicine, aiming to uncover the factors that either hinder or enable its successful adoption. The healthcare sector is no stranger to innovation, but the assimilation of AI technologies into daily practice comes with unique challenges. Family physicians, who are on the frontline of patient care, need to navigate these challenges while preserving the core principles of healthcare: personalized, compassionate, and patient-focused treatment. By investigating the barriers, the obstacles that family medicine practitioners encounter when considering AI integration can be identified. These may include technical limitations, data privacy concerns, regulatory hurdles, and resistance to change. Understanding these challenges is fundamental in devising strategies to overcome them and unlock AI's potential. In addition, the study examines the facilitators that drive AI adoption within family medicine. Factors such as access to patient data, technological advancements, clinical decision support tools, and regulatory support play pivotal roles in smoothing the path toward AI integration. Recognizing these enablers allows healthcare providers to leverage AI effectively for enhanced patient care.

## Materials and methods

This study adopted a cross-sectional survey design. 

Recruitment and sampling

As the study focused on family medicine, the participants included physicians working in family medicine from public hospitals and primary care centers. As participants were purposively recruited from the selected hospitals, convenience, and purposive sampling techniques were adopted [16]. The inclusion criteria included physicians who had a minimum of three months of practice and have been using or aware of AI-powered solutions virtual assistants for not less than three months.

Instruments

The survey questionnaire is divided into two sections. The first section focuses on collecting demographic information related to age, gender, and experience with AI-assisted technologies. The second section focuses on collecting the data on AI technology influencing factors. This study has adopted four factors including performance expectancy (four items), effort expectancy (three items), social influence (three items), and facilitating conditions (four items) from [17,18]. In addition, behavioral intention (three items) was adopted from [19]. In addition, three factors including perceived privacy risks (PPR) (four items), trust (four items), and personal innovativeness (PI) (four items) were adopted from [15]. Furthermore, ethical concerns (EC) (five items) were adopted from [20], and facilitators (12 items) from [21-25]. The questionnaire was designed using Google Forms, by creating a link to access the survey. A pilot study was conducted with 14 physicians, and the data was analyzed. Cronbach alpha was calculated for all items and was observed to be greater than 0.7, indicating good internal consistency [26].

Ethical considerations

All the participants were fully informed about the study through an information sheet attached to the invitation email. Informed consent was taken from all the participants using a check button, before starting the survey. The participation was voluntary and the participants were assured of their anonymity and their rights with respect to the data. Ethical approval was received from the Ethics Committee of Imam Abdulrahman Bin Faisal University.

Data collection

A participant's information sheet is attached along with the invitation email (containing a survey link), explaining the rights of the participants, and forwarded to all the physicians who agreed to participate in the survey. A total of 216 family physicians participated in the survey. However, 178 of them responded and 21 responses were incomplete. After cleaning the data, a total of 157 family physician's responses were considered for data analysis.

Data analysis

To attain the objectives of the research, the researcher utilized IBM SPSS Statistics for Windows, Version 24 (Released 2016; IBM Corp., Armonk, New York, United States) for analyzing the data. Descriptive statistics was used to characterize the participants’ demographic data. Mean scores (out of rating 5) for different factors were calculated. In addition, the two-sample t-test with unequal variances and Pearson correlation coefficient were used for analyzing the data. Furthermore, Person correlation coefficients were used for comparing the relationship between various factors.

## Results

As shown in Table 1, a total of 157 family physicians participated in the study, with appropriate representation of both genders (51.6% males and 48.4% females). Among the participants, 68.1% were aged below 41 years and 31.9% aged 41 or more years. The majority of the participants had master's or a higher degree.

**Table 1:** Participant's demographics

Variable		N	Relative frequency
Age (in years)	18-30	38	24.2%
	31-40	69	43.9%
	41-50	35	22.3%
	51-60	15	9.6%
Gender	Male	81	51.6%
	Female	76	48.4%
Education	Bachelor’s degree	29	18.5%
	Master’s degree or higher	128	81.5%

**Figure 1:**
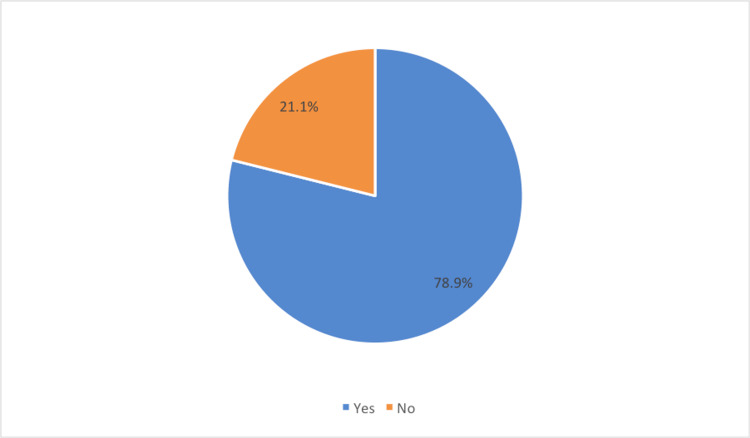
Usage of AI assistants by the participants

Among the total participants, 78.9% have used AI-enabled assistants for treatment and decision support as shown in Figure 1.

**Figure 2:**
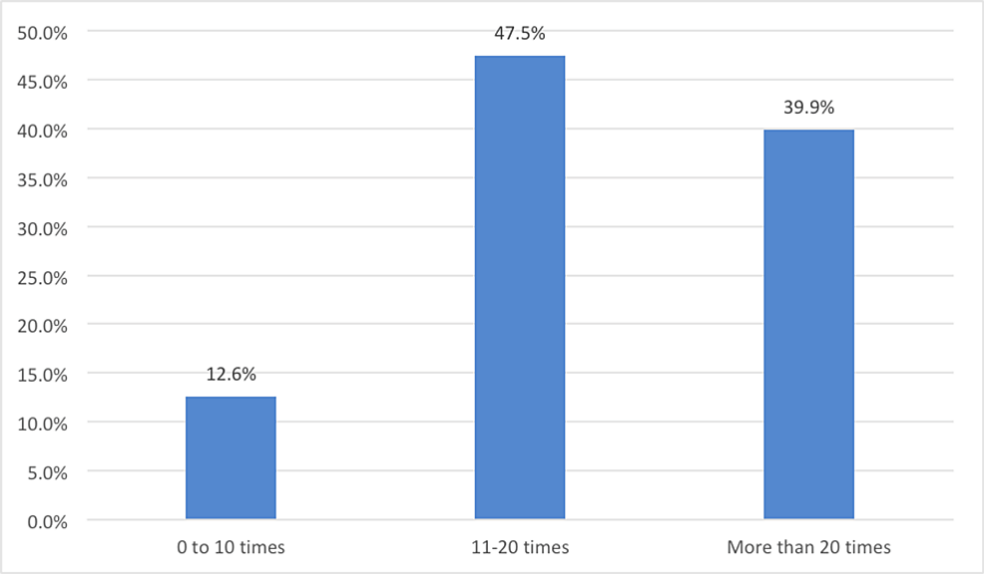
Frequency of the use of AI assistants by participants in the previous month (N=124)

Out of the 78.9% (124 participants) who used AI-enabled assistants, 47.5% used 11-20 times and 39.9% used more than 20 times in the last month indicating normal to high usage rates (Figure 2).

Family physicians' acceptance of AI-enabled assistants is influenced by several factors, as indicated by mean ratings in Table 2. Notably, they find AI assistants relatively easy to use, as reflected by a high mean rating for EE. Social influence (SI) and facilitating conditions (FC) also play moderately positive roles, suggesting that the opinions of colleagues and the presence of favorable conditions contribute to acceptance. Trust is another vital factor, with a moderate level of trust (Trust) seen in AI technology. However, there are notable concerns, such as PPR and EC, which received lower mean ratings, indicating that physicians have reservations about the privacy implications and ethical aspects of AI-enabled assistants. Nevertheless, the overall picture suggests a moderately positive attitude among family physicians, with BI indicating their willingness to embrace AI technology and the facilitator factor contributing to a supportive environment for its adoption.

**Table 2:** Mean ratings of various factors influencing acceptance of AI-enabled assistants

Factors	Mean (μ)
Performance expectancy (PE)	3.4
Effort expectancy (EE)	3.85
Social influence (SI)	3.64
Facilitating conditions (FC)	3.77
Behavioral intention (BI)	3.58
Trust	3.57
Perceived privacy risk (PPR)	2.82
Personalized innovativeness (PI)	3.45
Ethical concerns (EC)	2.44
Facilitators	3.31

Family physicians' acceptance of AI-enabled assistants is influenced by various facilitators, as revealed by mean ratings in Table 3. Access to data stands out as the most significant facilitator, with a high mean rating, highlighting the crucial role of data availability in AI acceptance. The availability of increased computing power is also viewed positively, emphasizing the importance of computational capabilities. Digital health technologies and patient engagement are moderately influential factors, indicating the relevance of technology integration and patient involvement in healthcare. However, regulatory support and interoperability standards received relatively low mean ratings, suggesting that family physicians may perceive shortcomings in the existing regulatory framework and interoperability in AI adoption. This data underscores the importance of addressing regulatory concerns and enhancing data accessibility to promote the acceptance of AI-enabled assistants by family physicians in the healthcare sector.

**Table 3:** Mean ratings of various facilitators

Facilitators	Mean (μ)
Access to data	4.33
Advancements in machine learning	3.23
Increased computing power	3.92
Digital health technologies	3.58
Regulatory support	2.29
Interoperability standards	2.71
Collaboration and education	2.76
Patient engagement	3.57
Cost-efficiency	3.34
Clinical decision support	3.08
Telemedicine	3.78
Research and development	3.15

The data provided in Table 4 reveals a comparative analysis of the acceptance factors for AI-enabled assistants among male and female respondents. In the case of PE, females showed a slightly higher mean rating than males, although the difference was not statistically significant (p = 0.08), suggesting a marginal gender-based trend. EE yielded comparable mean ratings between both genders, indicating no substantial difference in perceived ease of use. Likewise, for SI, both males and females reported similar mean scores, and the p-value (p = 0.49) substantiates the lack of a significant gender-related contrast in this regard. FC, BI, trust, PI, and EC also demonstrated minimal variations between genders with non-significant p-values. However, the factor of PPR displayed a more pronounced gender distinction, with females reporting significantly lower mean ratings than males (p = 0.01), signifying that females may have more privacy concerns regarding AI-enabled assistants. In summary, while there is some subtle gender-based disparities in the acceptance factors, they generally do not show strong distinctions between male and female respondents.

**Table 4:** Differences of participants’ perceptions related to factors influencing acceptance of AI-enabled assistants by gender * Statistically significant difference; df: degrees of freedom; SD: standard deviation; PE: performance expectancy; EE: effort expectancy; SI: social influence; FC: facilitating conditions; BI: behavioral intention; PPR: perceived privacy risk; PI: personalized innovativeness; EC: ethical concerns

Factors	Gender	N	Mean	SD	df	T-value	p-value
PE	Male	81	3.3	.86	152	1.35024	.0894
	Female	76	3.51	.98			
EE	Male	81	3.88	.45	151	.45322	.3255
	Female	76	3.82	.56			
SI	Male	81	3.65	.71	153	.00784	.4968
	Female	76	3.64	.79			
FC	Male	81	3.79	.44	141	.19037	.4246
	Female	76	3.81	.71			
BI	Male	81	3.56	.82	154	.27675	.3911
	Female	76	3.61	.61			
Trust	Male	81	3.61	.49	155	.50162	.3083
	Female	76	3.54	.47			
PPR	Male	81	2.94	.41	151	2.24369	.0131*
	Female	76	2.7	.51			
PI	Male	81	3.39	.47	154	1.21413	.1132
	Female	76	3.52	.49			
EC	Male	81	2.41	.81	155	.55469	.2899
	Female	76	2.48	.59			
Facilitators	Male	81	3.32	.17	146	.53715	.2959
	Female	76	3.28	.25			

The data analysis from Table 5 reveals notable variations in how different age groups perceive factors influencing the acceptance of AI-enabled assistants. For PE, respondents aged 40 years and below demonstrated a significantly higher mean rating compared to those over 40, signifying a stronger belief in the performance potential of AI technology among the younger group. A similar trend was observed for EE, with the younger respondents perceiving AI assistants as easier to use. The age-related differences were highly significant for both PE and EE. SI also displayed a significant variation, with the younger group attributing more importance to it than the older group. FC and BI were rated higher by the younger group, and trust had a significant difference with younger respondents exhibiting greater trust in AI-enabled assistants. PPR showed a substantial age-related distinction, with the older group expressing higher privacy concerns. PI exhibited a small but significant difference, with the younger group showing more openness to personalized innovations. Lastly, EC were significantly lower for the older group, indicating a higher level of concern regarding ethical issues. Facilitators, such as access to data and technological advancements, did not show significant differences between the two age groups.

**Table 5:** Differences of participants’ perceptions related to factors influencing acceptance of AI-enabled assistants by age * Statistically significant difference; df: degrees of freedom; SD: standard deviation; PE: performance expectancy; EE: effort expectancy; SI: social influence; FC: facilitating conditions; BI: behavioral intention; PPR: perceived privacy risk; PI: personalized innovativeness; EC: ethical concerns

Factors	Age	N	Mean	SD	df	T-value	p-value
PE	<=40 years	107	3.92	.31	87	15.7441	< .0001*
	> 40 years	50	2.28	.40			
EE	<=40 years	107	4.02	.36	76	4.1219	< .0001>
	> 40 years	50	3.5	.63			
SI	<=40 years	107	3.76	.64	82	2.3029	.0119*
	> 40 years	50	3.41	.91			
FC	<=40 years	107	3.93	.46	83	3.05105	.0015*
	> 40 years	50	3.52	.66			
BI	<=40 years	107	3.78	.65	100	4.5694	< .0001*
	> 40 years	50	3.16	.59			
Trust	<=40 years	107	3.75	.42	99	5.2924	< .0001*
	> 40 years	50	3.18	.39			
PPR	<=40 years	107	2.68	.42	93	3.8616	.0001*
	> 40 years	50	3.13	.45			
PI	<=40 years	107	3.53	.47	96	2.1782	.0159*
	> 40 years	50	3.28	.45			
EC	<=40 years	107	2.63	.77	138	5.02001	< .0001*
	> 40 years	50	2.04	.33			
Facilitators	<=40 years	107	3.34	.21	94	1.4569	.0742
	> 40 years	50	3.22	.22			

Overall, these findings suggest that younger individuals tend to have more positive attitudes and expectations towards AI-enabled assistants, while older individuals may have greater concerns and reservations, particularly in terms of privacy and ethics. The correlation matrix in Table 6 reflects weak positive correlations between all the factors and BI, except social influence and PPR, which are negatively correlated with behavioral intentions to accept AI-enabled assistants. In addition, PPR was identified to be negatively correlated with all other factors, indicating it as the major barrier in AI-assistant acceptance.

**Table 6:** Correlation matrix PE: performance expectancy; EE: effort expectancy; SI: social influence; FC: facilitating conditions; BI: behavioral intention; PPR: perceived privacy risk; PI: personalized innovativeness; EC: ethical concerns

	PE	EE	SI	FC	Trust	PPR	PI	EC	BI
PE	1								
EE	0.432	1							
SI	0.227	0.136	1						
FC	0.240	0.177	0.164	1					
Trust	0.398	0.277	0.182	0.324	1				
PPR	-0.243	-0.140	-0.112	-0.269	-0.152	1			
PI	0.177	0.165	0.142	0.067	0.013	-0.107	1		
EC	0.217	0.135	-0.036	0.121	0.208	-0.123	0.046	1	
BI	0.287	0.026	-0.004	0.228	0.254	-0.255	0.150	0.238	1

## Discussion

The purpose of this study is to explore the factors that affect the adoption of AI-enabled assistants by family physicians in their tasks such as treatment and decision-making. More than two-thirds of the participants used AI assistants, with the majority among them using them more than ten times in the previous month indicating gradual penetration of AI assistants in family medicine. Similar results were identified in other studies focusing on specialties like cardiology and other healthcare departments on the use of AI assistants in clinical decision-making [27-30]. The results from the survey analysis have indicated various barriers and facilitators, which had a differential impact on the participants. Assessing the positive influence of the factors, it was observed that EE and facilitating conditions were identified to be strong influence factors. These findings indicate that the perceived ease of use or convenience and usability of AI assistants among the participants is high, which can also be correlated with facilitating conditions that provide support in using the assistants in similar studies [31,32].

Family medicine practitioners have access to a wealth of patient data, including electronic health records, medical histories, and diagnostic test results, which is essential for training AI models and making accurate predictions [33-35]. In addition, availability of high-performance computing resources enables the rapid processing of large datasets, making it feasible to develop and deploy AI systems in real-time clinical settings [36]. Also, the rise of telemedicine has created opportunities for AI-powered virtual health assistants and remote monitoring systems, enabling family physicians to extend their reach and provide care to patients regardless of geographic location [37]. Accordingly, the findings in this study identified access to data, increased computing power, and telemedicine as major facilitators. However, regulatory support and interoperability standards were poorly rated indicating these factors as barriers [38,39].

While there are no major differences between the participant's genders in relation to the factors influencing acceptance of AI assistants, significant differences (p < .05) were identified between participants' age groups. Overall, these findings suggest that younger individuals tend to have more positive attitudes and expectations toward AI-enabled assistants, while older individuals may have greater concerns and reservations, particularly in terms of privacy and ethics [40,41]. Furthermore, PE and EE are moderately positively correlated, indicating that as performance expectations rise, so does the perception of ease of use. This suggests that physicians who anticipate better performance from AI technology also tend to find it easier to use. Additionally, PE and trust exhibit a moderate positive correlation, signifying that physicians with higher performance expectations also tend to have higher levels of trust in AI-enabled assistants. On the other hand, PPR is negatively correlated with several factors. PPR has negative correlations with PE, EE, SI, FC, and Trust. This implies that as perceived privacy risk increases, acceptance-related factors such as performance expectations, ease of use, social influence, environmental support, and trust tend to decrease. Family physicians who are more concerned about privacy may, therefore, have lower acceptance of AI-enabled assistants. In summary, these correlations highlight the complex interplay between different factors and underscore the importance of addressing privacy concerns to promote acceptance among family physicians.

Implications

This study reveals crucial insights with both theoretical and practical implications. The study sheds light on the role of age and gender in shaping family physicians' acceptance of AI technology, emphasizing the need for tailored educational and training programs to bridge knowledge gaps and address privacy concerns. It underscores the significance of addressing barriers, such as ethical concerns and perceived privacy risks, while capitalizing on facilitators, including improved access to data and increased computing power, to promote AI adoption effectively. The study also highlights the pivotal role of trust in AI acceptance, calling for transparent communication and measures to build trust among healthcare providers. These findings provide a valuable foundation for healthcare institutions to develop targeted strategies for AI adoption, ensuring that family physicians can harness the potential of AI-enabled assistants to enhance patient care and clinical decision-making in family medicine. Continuous evaluation and adaptation of these strategies will be essential in the dynamic landscape of AI adoption in healthcare.

Limitations

While this study offers valuable insights into the factors influencing the adoption of AI-enabled assistants in family medicine, it is essential to acknowledge several limitations. First, the research was conducted with a specific focus on family physicians, and the findings may not be directly transferable to other healthcare specialties or settings. The study's sample size, although substantial, may not fully represent the diverse population of family physicians, potentially introducing selection bias. Moreover, the study relied on self-reported data, which can be subject to response bias and may not always reflect actual behavior or attitudes accurately. Additionally, the research is based on cross-sectional data, which limits the ability to establish causality or assess changes in attitudes over time. The use of a survey questionnaire for data collection might not capture the nuances and in-depth insights that qualitative methods could provide. Finally, while the study explores the impact of various factors on AI acceptance, it does not delve into the potential mediating or moderating effects among these factors, which could provide a deeper understanding of the dynamics at play. Considering these limitations, future research should consider longitudinal and mixed-methods approaches to provide a more comprehensive view of AI adoption in family medicine, accounting for the evolving nature of this field and the diverse perspectives of healthcare professionals.

## Conclusions

In conclusion, this empirical study offers valuable insights into the acceptance of AI-enabled assistants in family medicine, shedding light on the multifaceted factors that influence family physicians' attitudes and intentions regarding AI adoption. The findings underscore the importance of addressing key barriers, such as ethical concerns and perceived privacy risks, while capitalizing on facilitators like improved access to data and increased computing power to facilitate the integration of AI technology effectively. The role of trust in AI adoption emerges as a pivotal element, highlighting the need for transparent communication and measures to build trust among healthcare providers. Additionally, the study's exploration of age and gender disparities in AI acceptance emphasizes the importance of tailored educational and training programs to bridge knowledge gaps and address privacy concerns among different demographic groups. These insights have significant implications for healthcare institutions striving to harness AI's potential in family medicine, enhancing patient care and clinical decision-making. The dynamic nature of AI adoption in healthcare calls for ongoing evaluation and adaptation of strategies to keep pace with technological advancements and evolving healthcare needs. As AI continues to shape the future of healthcare, understanding these factors becomes increasingly vital for healthcare professionals and institutions committed to delivering high-quality, patient-centered care.
